# Integrated Omics Analysis Uncovers the Culprit behind Exacerbated Atopic Dermatitis in a Diet-Induced Obesity Model

**DOI:** 10.3390/ijms25084143

**Published:** 2024-04-09

**Authors:** You Mee Ahn, Jeeyoun Jung, So Min Lee

**Affiliations:** KM Science Research Division, Korea Institute of Oriental Medicine, Daejeon 34054, Republic of Korea; 2hyunm@kiom.re.kr (Y.M.A.); dasonya@kiom.re.kr (S.M.L.)

**Keywords:** atopic dermatitis, obesity, lipidomics, transcriptomics, arachidonic acid, phospholipid metabolism

## Abstract

Atopic dermatitis (AD), a chronic inflammatory skin disease, is exacerbated by obesity, yet the precise linking mechanism remains elusive. This study aimed to elucidate how obesity amplifies AD symptoms. We studied skin samples from three mouse groups: sham control, AD, and high-fat (HF) + AD. The HF + AD mice exhibited more severe AD symptoms than the AD or sham control mice. Skin lipidome analysis revealed noteworthy changes in arachidonic acid (AA) metabolism, including increased expression of *pla2g4*, a key enzyme in AA generation. Genes for phospholipid transport (*Scarb1*) and acyltransferase utilizing AA as the acyl donor (*Agpat3*) were upregulated in HF + AD skin. Associations were observed between AA-containing phospholipids and skin lipids containing AA and its metabolites. Furthermore, imbalanced phospholipid metabolism was identified in the HF + AD mice, marked by excessive activation of the AA and phosphatidic acid (PA)-mediated pathway. This imbalance featured increased expression of *Plcb1*, *Plcg1*, and *Dgk* involved in PA generation, along with a decrease in genes converting PA into diglycerol (DG) and CDP-DG (*Lpin1* and *cds1*). This investigation revealed imbalanced phospholipid metabolism in the skin of HF + AD mice, contributing to the heightened inflammatory response observed in HF + AD, shedding light on potential mechanisms linking obesity to the exacerbation of AD symptoms.

## 1. Introduction

Atopic dermatitis (AD) is a chronic inflammatory disease with a complex pathophysiology ranging from an imbalanced type 1 helper (Th1)/type 2 helper (Th2) ratio to defective innate immune system and skin barrier function [1]. This imbalance extends its influence to epithelial cells, impacting epithelial polarization through a transcription factor network [2]. The increasing prevalence of both AD and obesity can be attributed to various factors, such as Western-style diets, urbanization, and changes in family structures [1,3,4,5]. Extensive research has explored the association between obesity and AD, with meta-analyses confirming this link [6,7,8]. Experimental models have shed light on the connections between AD and obesity, implicating adipokines like leptin and adiponectin in both conditions [7]. Additionally, impaired lymphatic function in obesity has been proposed to exacerbate dermatitis hypersensitivity [8]. Furthermore, prostaglandins, pivotal in type 2 diseases, require consideration [9]. The link between AD and obesity gains further support from evidence of tissue-based low-grade inflammation in obese individuals without comorbidities [10]. However, the precise mechanisms underlying how obesity exacerbates AD, and the mediators involved, remain incompletely understood.

High-fat diets can induce alterations in plasma lipid profiling, impacting lipid metabolism in various organs [11]. Given the essential role of lipids in maintaining skin health, these changes in blood lipid metabolism are likely to influence skin lipid metabolism, especially in conditions like AD [12]. Consequently, investigating both skin lipid metabolism and circulating lipid profiles may reveal crucial mechanisms contributing to the exacerbation of AD associated with obesity.

Lipidomics, in conjunction with transcriptomics, has shed light on disease-related alterations in the lipidome, offering insights into overall gene expression and differentially expressed genes (DEGs) [13,14]. In the context of atopic dermatitis (AD), lipid mediators like prostaglandins and specialized pro-resolving mediators (SPMs) play pivotal roles in regulating inflammation and immune responses [15]. Understanding the involvement of these mediators enhances our comprehension of AD pathogenesis and offers potential therapeutic targets, especially in the context of obesity-induced metabolic disturbances. To explore these mechanisms, we developed AD models using dinitrochlorobenzene (DNCB) treatment in normal-weight mice fed a chow diet and obese mice fed a high-fat diet (HF), with 60% of the calories derived from lipids. Integrating transcriptomic and lipidomic analyses, our aim was to unravel the underlying molecular mechanisms driving AD exacerbation in the context of obesity. Furthermore, we investigated the altered relationship between circulating lipids and skin lipids induced by the HF diet, aiming to reveal the underlying mechanisms contributing to changes in AD-exacerbating factors.

## 2. Results

### 2.1. High-Fat-Diet-Induced Obesity Exacerbates DNCB-Induced AD-Like Lesions

**Figure 1:**
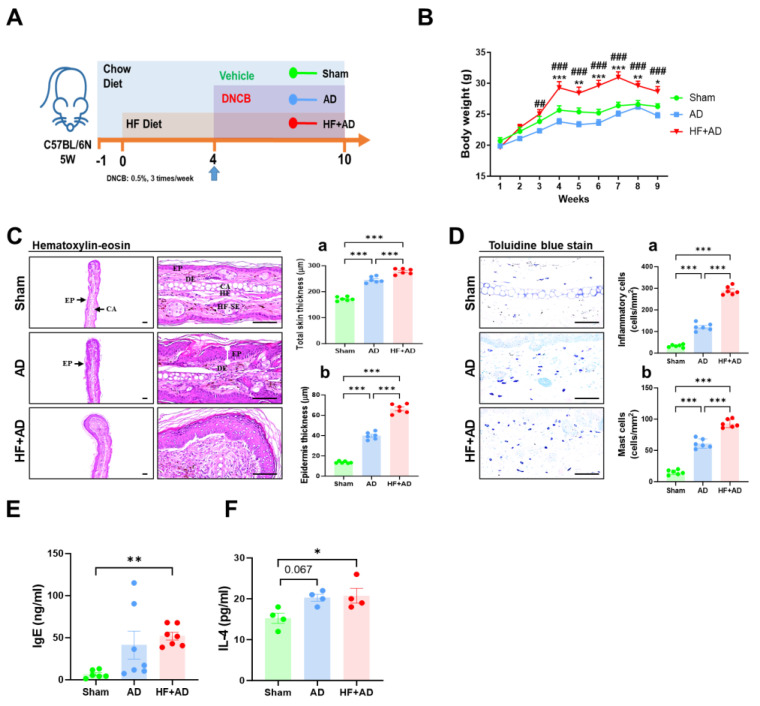
HF diet–induced obesity exacerbates DNCB–induced AD–like lesions. (**A**) Timeline of the experiments. (**B**) Body weight changes (sham, *n* = 6; AD, *n* = 7; HF + AD, *n* = 7), * *p* < 0.05, ** *p* < 0.01, *** *p* < 0.001 vs. sham; ^##^ *p* < 0.01, and ^###^ *p* < 0.001 vs. AD using two–way ANOVA. (**C**) Measurement of the thickness of total ear skin (**a**) and dermis (**b**) using hematoxylin and eosin staining microphotographs (*n* = 6 for all groups). (**D**) Measurement of levels of inflammatory cells (**a**) and mast cells (**b**) using toluidine blue staining (n = 6 for all groups). *** *p* < 0.001 estimated using one–way ANOVA with Tukey’s multiple comparisons tests. (**E**) Plasma IgE (sham, *n* = 6; AD, *n* = 7; HF + AD, *n* = 7), with ** *p* < 0.01 compared to the sham group according to Kruskal–Wallis test. (**F**) Plasma IL–4 (*n* = 4 for all groups). * *p* < 0.05 estimated using one–way ANOVA with Tukey’s multiple comparisons tests. HF, high–fat; DNCB, 2,4–dinitrochlorobenzene; AD, atopic dermatitis; DE, dermis; HF–SE, hair follicle–sebaceous gland; EP, epidermis; CA, ear cartilage; IgE, immunoglobulin E; IL–4, interleukin–4; IL–13, interleukin–13; ANOVA, analysis of variance.

The development of an obesity–AD comorbidity model is schematically shown in Figure 1A. The HF + AD group had significantly higher body weights than the AD group starting from two weeks to the end of the experiment. The AD group exhibited lower body weights than the sham group starting from 4 to 8 weeks (Figure 1B).

Histopathological examination revealed marked thickening of the skin tissues in the AD and HF + AD mice compared with the sham mice, as evidenced by hyperplasia of epidermal keratinocytes and dermal infiltration of inflammatory and mast cells (Figure 1C). The skin thickening was more severe in the HF + AD group than in the AD group (Figure 1D; *p* < 0.001 for all comparisons). The dorsal skin exhibited similarities to the ear skin as well. Plasma IgE levels were also increased in the order of sham, AD, and HF + AD (Figure 1E), along with changes in Th2 cytokines such as IL-4 (Figure 1F). These changes fell within the detection limits of the utilized assay, as indicated by the LLODs and HLODs in Table 1. These results implied that there were more severe AD symptoms exhibited by the HF + AD mice than those for the AD and sham mice.

### 2.2. Untargeted Skin Lipidomics Demonstrates Changes in Lipid Metabolism in HF + AD Mice

**Figure 2:**
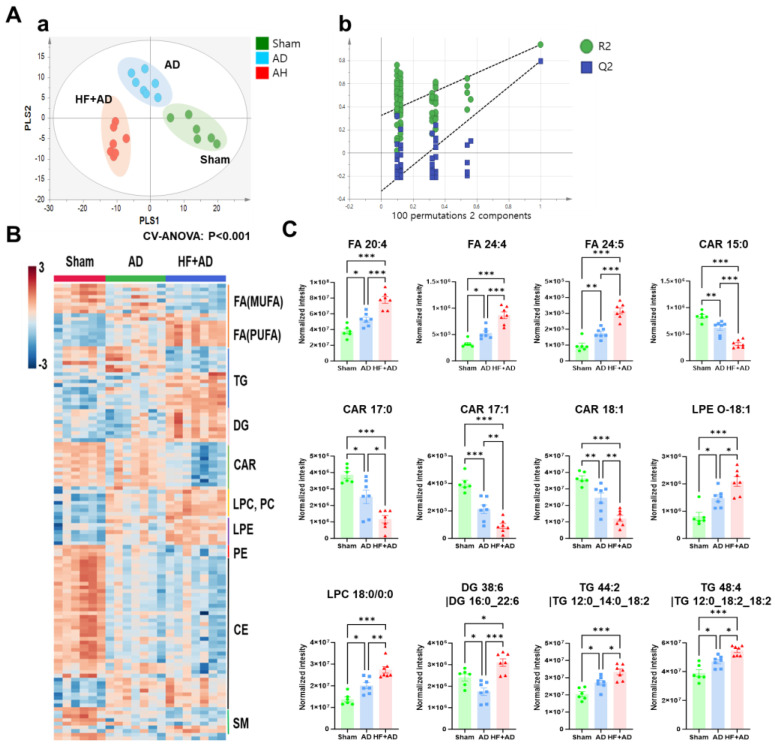
The untargeted lipidomics analysis indicates the differences in skin lipid metabolism among the sham, AD, and HF + AD groups. (**A**) PLS–DA score plot of skin lipid profiles obtained from sham, AD, and HF + AD mice (**a**). Validation of the PLS–DA model based on the 999–repeated permutation (**b**). (**B**) The pattern search analysis focused on significant skin lipids in the PLS–DA model based on the variable–importance–on–projection (VIP) values (given pattern: 1–sham, 2–AD, 3–HF + AD). (**C**) Skin lipid markers selected by the combination of VIP, pattern search, and one–way ANOVA tests. * *p* < 0.05, ** *p* < 0.01, and *** *p* < 0.001 estimated using one–way ANOVA with Tukey’s multiple comparisons tests. Number of experiments: sham, *n* = 6; AD, *n* = 7; HF + AD, *n* = 7. HF, high–fat; AD, atopic dermatitis; FA, fatty acid; MUFA, Monounsaturated fatty acid; PUFA, Polyunsaturated fatty acid; TG, triglyceride; DG, Diglyceride; CAR, carnitine; LPC, Lysophosphatidylcholine; PC, Phosphatidylcholine; LPE, lysophosphatidylethanolamine; PE, Phosphatidylethanolamine; CE, Ceramide; SM, Sphingomyelin; VIP, variable importance on projection; ANOVA, analysis of variance.

To confirm the changes in lipid metabolism in the skin of the HF-diet-induced obesity AD model, we performed unbiased lipidomics analysis on skin samples. As shown in Figure 2A, the score plot of PLS-DA using the untargeted skin lipidome data shows different lipid patterns among the sham, AD, and HF + AD groups. This model was validated by a 999-repeated permutation test.

Metabolites that significantly contributed to model establishment (with a variable importance on projection score > 1) and were pattern-analysis-matched are shown in Figure 2B. Specifically, fatty acids showed different patterns in accordance with the level of saturation. The abundance of mono-unsaturated fatty acids decreased, whereas that of polyunsaturated fatty acids increased in the following order: sham, AD, and HF + AD. The abundance of diglyceride (DG), lysophosphatidylcholine (LPC), phosphatidylcholine (PC), and lysophosphatidylethanolamine (LPE) increased in the following order: sham, AD, and HF + AD. Carnitine (CAR) and ceramide levels in the AD and HF + AD mice were lower than those in the sham mice (Figure 2B).

Of these lipid metabolites, 12 exhibited statistical significance in all-pair comparisons (Figure 2C). The levels of fatty acid (FA) 20:4, 24:4, and 24:5; LPE O-18:1; LPC 18:0; DG 38:6; and TG 44:2 and 48:4 were the highest in the HF + AD group. In contrast, the levels of CARs, such as CAR 15:0, 17:0, 17:1, and 18:1, were the lowest in the HF + AD group. The metabolic pathway enrichment analysis of these 12 metabolites indicated significant changes in arachidonic acid (AA) metabolism and the regulation of inflammatory mediator transient receptor potential channels (*p* < 0.05) in the HF + AD group compared to the other groups (Table 1).

### 2.3. Transcriptomic Analysis Highlights Distinctions in Pathways Associated with Atopic Immune Responses and Lipid Metabolism between AD and HF + AD

To identify the underlying cause of the differences in AD symptoms and lipid patterns between the AD and HF + AD groups, we conducted transcriptomic analysis, obtaining skin samples from the AD and HF + AD mice.

**Figure 3:**
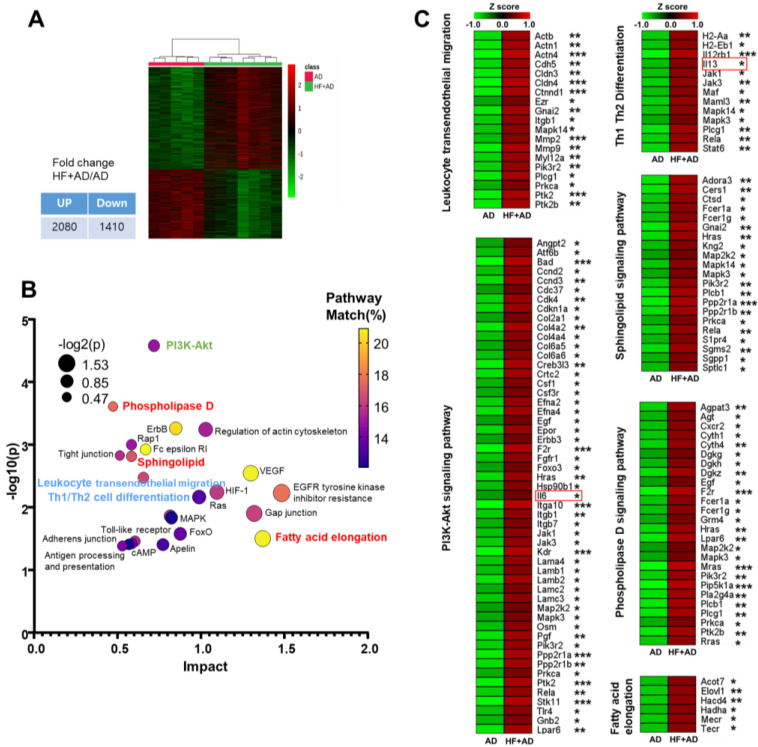
Transcriptomics reveals the differences in pathways related to the atopic immune responses and lipid metabolism between AD and HF + AD. (**A**) Heat map of RNA–seq data for differentially regulated genes in skin lesions (*p* < 0.05). (**B**) KEGG pathway enrichment analysis. (**C**) The altered genes in leukocyte transendothelial migration, PI3K–Akt signaling pathway, Th1 and Th2 cell differentiation, sphingolipid signaling pathway, phospholipase D signaling pathway, and fatty acid elongation. Number of experiments: AD, *n* = 7; HF + AD, *n* = 7. * *p* < 0.05, ** *p* < 0.01, and *** *p* < 0.001 as determined via unpaired Student’s *t*-test. HF, high–fat; AD, atopic dermatitis; PI3K, phosphoinositide–3–kinase; Th1, T helper type 1 cell; Th2, T helper type 2 cell; ANOVA, analysis of variance.

In total, 2080 and 1410 genes were differentially up- and down-regulated in the HF + AD group compared with their expression in the AD group, respectively (*p* < 0.05; Figure 3A). A pathway enrichment analysis using 2080 of the up-regulated genes showed that pathways related to atopic immune responses such as Th1 and Th2 cell differentiation and leukocyte transendothelial migration were more significantly up-regulated in the HF + AD group than in AD mice. Specifically, the mRNA expression of IL-13, a representative Th2 cytokine [16], was higher in the HF + AD mice than in the AD mice. In addition, the PI3-Akt signaling pathway exhibited the highest log10(p) value (Figure 3B); this pathway is linked to the activation of peripheral T cells in AD patients, resulting in the secretion of cytokines such as IL-6 and IL-10 [17]. In line with this, we also verified that there was significantly higher mRNA expression of IL-6 in the HF + AD mice than in the AD group. Furthermore, the pathways involved in lipid metabolism, such as the phospholipase D, sphingolipid, and the fatty acid elongation pathways, were also more significantly up-regulated in the HF + AD group. The genes involved in these enriched pathways are listed in Figure 3C.

### 2.4. The Enriched KEGG Pathway Network Reveals the Involvement of Phospholipase Activation in Mediating the Inflammatory Response in the Skin of HF + AD Mice

**Figure 4:**
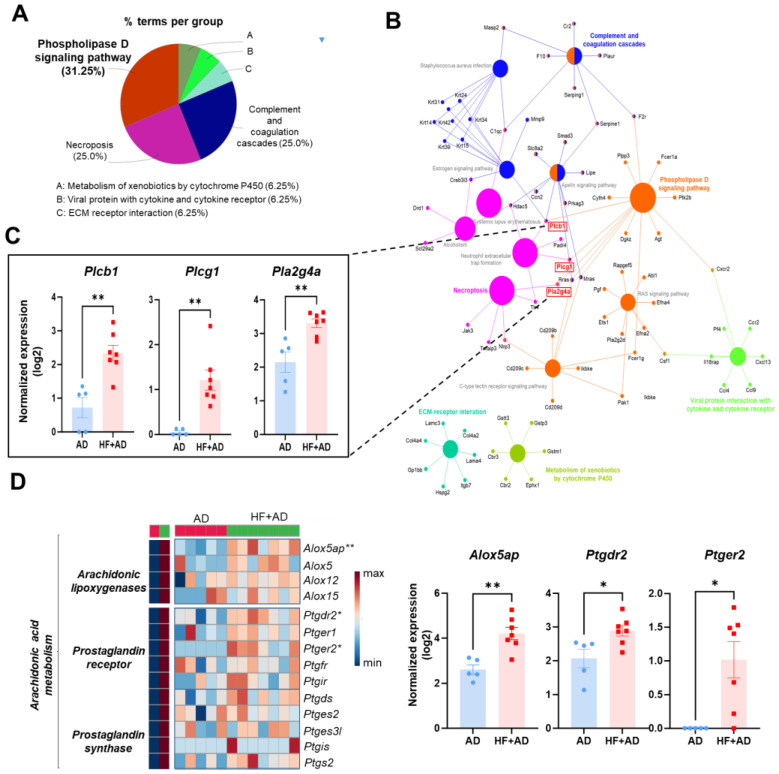
The enriched KEGG pathway network reveals the involvement of phospholipase activation in mediating the inflammatory response in the skin of HF + AD mice. (**A**) A pie chart with functional groups, including up-regulated genes in the HF + AD group. (**B**) Functionally grouped networks based on KEGG pathway with terms presented as nodes linked based on k score level (>3). (**C**) The expression of *Plcb1*, *Plcg1*, and *Pla2g4a*. These genes are co–expressed in the phospholipase D signaling and necroptosis pathways (**D**) The heatmap of genes in arachidonic acid metabolism (**left**) and the changes in *Alox5ap*, *Ptgdr2*, and *Ptger2* (**right**) between AD and HF + AD. Number of experiments: AD, *n* = 5; HF + AD, *n* = 7. * *p* < 0.05 and ** *p* < 0.01, according to Mann–Whitney U test. HF, high–fat; AD, atopic dermatitis; KEGG, Kyoto encyclopedia of genes and genomes; *Plcg1*; phospholipase C gamma 1, *Plcb1*; phospholipase C beta 1, *Pla2g4a*; phospholipase A2 group IVA, *Alox5ap*; arachidonate 5–lipoxygenase-activating protein, *Ptgdr2*; prostaglandin D receptor 2, *Ptger2*; prostaglandin E receptor 2; ANOVA, analysis of variance.

ClueGO enrichment analysis using up-regulated genes with over 2-fold changes between AD and HF + AD (Figure 4A) indicated that the predominant enrichment pathways are as follows: phospholipase D pathway (31.25%), necroptosis (25%), complement and coagulation cascades (25%), viral protein infection with cytokine and cytokine receptor (6.25%), metabolism of xenobiotics by cytochrome P450 (6.25%), and ECM–receptor interaction (6.25%). Among these genes, those encoding phospholipase C, including phospholipase C gamma 1 (*Plcg1*) and phospholipase C beta 1 (*Plcb1*), the latter of which hydrolyzes phospholipids and generates two second messengers, inositol 1,4,5-triphosphate (IP3) and DG [18], and phospholipase A, specifically phospholipase A2 group member IVA (*Pla2g4a*), which converts phospholipids to AA and lysophospholipids, were found to be co-expressed in two major pathways: the phospholipase D signaling and necroptosis pathways. (Figure 4B,C).

We identified significant alterations in the expression of mRNA related to the AA inflammatory pathway, such as arachidonate 5-lipoxygenase-activating protein (*Alox5ap*)*,* prostaglandin E receptor 2 (*Ptger2*), and prostaglandin D receptor 2 (*Ptgdr2*), in the HF + AD groups compared with the AD group (Figure 4D).

### 2.5. Circulating AA Correlated with the Changes in AA and Its Metabolites in Skin of HF + AD Mice

**Figure 5:**
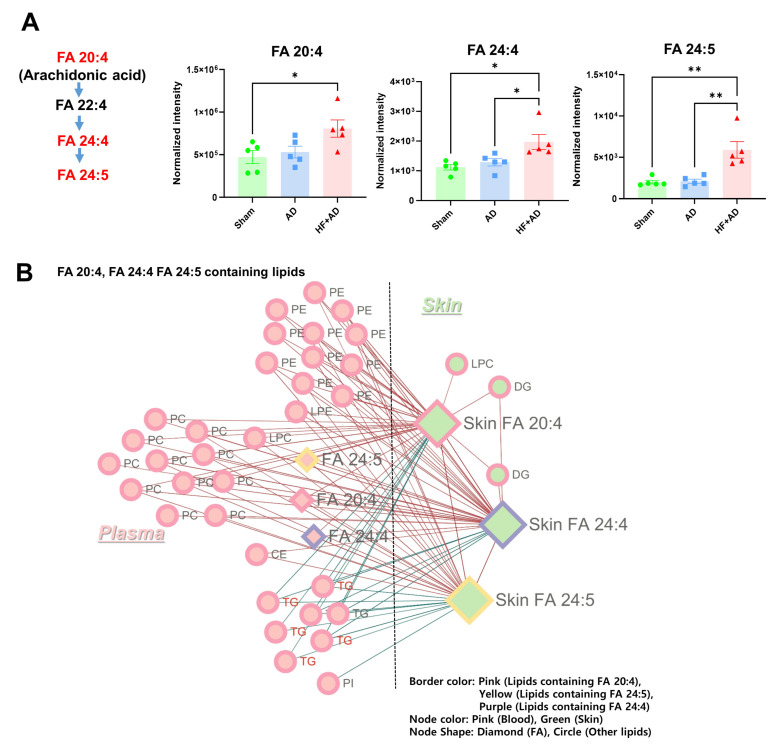
The levels of circulating AA are associated with alterations in both AA and its metabolites in the skin of mice with HF + AD. (**A**) The plasma levels of AA (FA 20:4) and metabolites (FA 24:4 and FA 24:5). (**B**) The results of the correlation network analysis between AA and its metabolites in the plasma and skin. Number of experiments: sham, *n* = 5; AD, *n* = 5: HF + AD, *n* = 5. * *p* < 0.05 and ** *p* < 0.01, estimated using one–way ANOVA with Tukey’s multiple comparisons tests. AA, arachidonic acid; HF, high–fat; AD, atopic dermatitis; FA, fatty acid; PE, phosphatidylethanolamine; PC, phosphatidylcholine; LPE, lysophosphatidylethanolamine; DG, diglyceride; PI, phosphatidylinositol; ANOVA, analysis of variance.

We examined the relationship between AA and its metabolites in the plasma and skin. In the plasma of the HF + AD mice, the levels of AA (FA 20:4) exceeded those in the sham group, and the levels of metabolites (FA 24:4, and FA 24:5) were significantly higher in the HF + AD group than in the sham and AD groups (Figure 5A). Circulating FA 20:4, FA 24:4, and FA 24:5 also showed significant associations with FA 20:4, FA 24:4, and FA 24:5 in skin (Figure 5B).

The correlation network analysis provided a more detailed depiction of the relationships between AA and its metabolites in the plasma and skin (Figure 5B). The presence of phospholipids (PCs and PEs), containing FA 20:4, FA 24:4, and FA 24:5, circulating in the blood, exhibited the positive associations with variations in lipids containing FA 20:4, FA 24:4, and FA 24:5 in the skin. However, TGs containing FA 20:4, FA 24:4, and FA 24:5 were negatively correlated with lipids containing FA 20:4, FA 24:4, and FA 24:5 in the skin.

Among skin lipids, FA 20:4 and FA 24:4 displayed notable correlations with specific lipid molecules such as DGs (DG 18:0_20:4; DG 18:1_20:4) and LPC (LPC 20:4) (Figure 5B), which may reflect the activation of phospholipase C and phospholipase A.

### 2.6. HF-Diet-Induced Obesity Altered the Expression of Lipid Transporter Genes in Skin of DNCB-Induced AD-like Lesions

**Figure 6:**
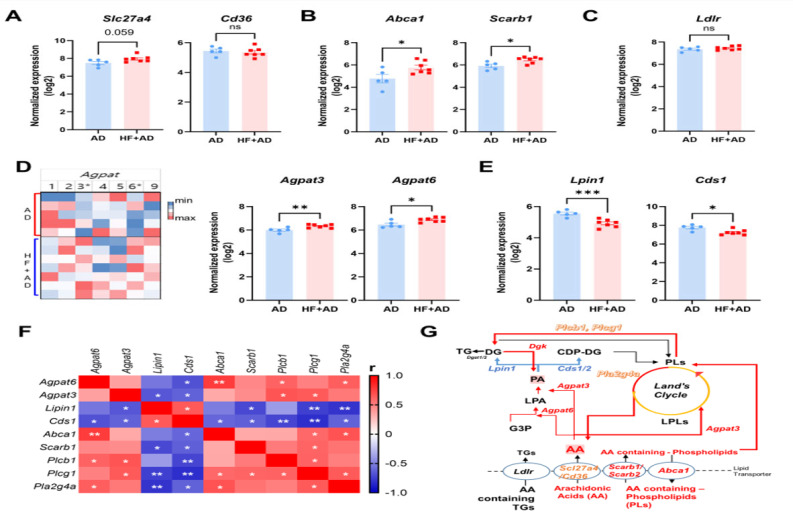
Alterations in the expression of genes related to lipid transport, the phospholipid de novo synthesis pathway, and the phospholipid remodeling pathway lead to an imbalance in phospholipid metabolism and an increase in AA levels in the skin of HF + AD mice. The expression of genes involved in the transport of (**A**) fatty acids (A, *Slc27a4* and *Cd36*), (**B**) phospholipids (*Abca1* and *Scarb1*), and (**C**) triglycerides (*Ldlr*). (**D**) Heatmap of AGPAT Family (**left**) and expression of *Agpat3* and *Agpat6* (**right**) in phospholipid de novo synthesis and remodeling pathways. (**E**) The expression of mRNA involved in phospholipid and triglyceride de novo synthesis (*Lpin1* and *Cds1*) (**F**) The correlations between mRNA expression in phospholipid metabolism. (**G**) The imbalance of phospholipid metabolism in the skin lesions of HF + AD mice. Number of experiments: AD, *n* = 5; HF + AD, *n* = 7. * *p* < 0.05, ** *p* < 0.01, and *** *p* < 0.001; two groups were compared using a two–sided *t*-test or, for non–Gaussian distribution data, the Mann–Whitney U test. HF, high–fat; AD, atopic dermatitis; *Scl27a4*, solute carrier family 26 (fatty acid transporter) member 4; *Abca1*, ATP–binding cassette subfamily member 1; *Scarb1*, scavenger receptor class B member 1; *Ldlr*, low–density lipoprotein receptor; *Agpat3*, 1–acylglycerol–3–phosphate O–acyltransferase 3; *Agpat6*, 1–acylglycerol–3–phosphate O–acyltransferase 6; *Lpin1*, Lipin1; *Cds1*, CDP–diacylglycerol synthase 1; TG, triglyceride; DG, diglyceride; PA, phosphatidic acid; LPA, lysophosphatidic; PLs, phospholipids; AA, arachidonic acids; G3P, glycerol 3–phosphate.

To confirm the potential association between changes in circulating AA-containing lipids and skin phospholipid metabolism, we examined the expression of several lipid transporter genes (Figure 6A–C).

Among the lipid transporter genes, the expression of genes involved in the transport of fatty acids, namely, solute carrier family 26 (fatty acid transporter) member 4 (*Slc27a4*, *p* = 0.059), showed an increasing trend, but this was not statistically significant, whereas genes associated with the transport of phospholipids, such as ATP-binding cassette subfamily member 1 (*Abca1*, *p* < 0.05) and scavenger receptor class B member 1 *(Scar1*, *p* < 0.005), exhibited a significant upregulation in the HF + AD group (Figure 6A,B). However, the low-density lipoprotein receptor (*Ldlr*) did not show a significant change (Figure 6C).

### 2.7. Imbalanced Phospholipid Metabolism Induced by High-Fat-Diet-Aggravated AD in HF + AD Mice

Transported fatty acids and phospholipids undergo modifications via the phospholipid de novo synthesis pathway and remodeling pathway. Therefore, we explored the 1-acylglycerol-3-phosphate O-acyltransferase (*APGAT*) family, which is associated with acyltransferase activity in both the de novo synthesis and remodeling pathways (Figure 6D). The mRNA expression of 1-acylglycerol-3-phosphate O-acyltransferase 6 *(Agpat6*, *p* < 0.05, Figure 6D) in the initial step of the de novo pathway increased significantly [19], along with that of 1-acylglycerol-3-phosphate O-acyltransferase 3 *(Agpat3*, *p* < 0.01), which displayed acyltransferase activity in the phospholipid de novo pathway and remodeling cycle, leading not only to the production of phosphatidic acid (PA) but also the remodeling of LPLs into the AA-containing PLs [20].

The levels of the *Lipin1* (*Lpin1*, *p* < 0.001) and CDP-diacylglycerol synthase 1 (*Cds1*, *p* < 0.05), which play a role in generating new PLs and TGs by converting PA to DG and CDP-DG, were significantly lower in the HF + AD than AD mice (Figure 6E). The correlation matrix also revealed significant negative correlations between the expression of genes involved in converting PA into new PLs and TG and genes associated with each of the remaining pathways, including lipid transporter genes, phospholipase genes, and acyltransferase genes in phospholipid metabolism (Figure 6F).

Taken together, this study demonstrated that the breakdown of transported phospholipids, rather than utilizing newly synthesized phospholipids as substrates for phospholipases, and skewed phospholipid metabolism are associated with AD exacerbation in HF + AD mice.

## 3. Discussion

In the present study, we demonstrated that mice with HF-diet-induced obesity developed more severe AD than non-obese mice. In the HF + AD model, an elevation in the levels of leptin, known for its secretion from adipose tissue and induction of systemic inflammation [21], was noted. Furthermore, we observed pronounced hyperplasia in the atopic lesions, accompanied by increased migration of inflammatory cells, including mast cells, to the skin. Additionally, higher levels of IgE and IL-4 in the blood were observed in the HF + AD group than in the AD and control groups. In this regard, previous studies also [22,23] pointed out that leptin secreted in the state of obesity activates the innate and adaptive immune systems, which leads to the secretion of cytokines and promotes the migration of leukocyte migration, thus exacerbating the symptoms of AD. However, in this study, we conducted lipidomics and transcriptomics analyses focusing on the lipid metabolism of an HF + AD animal model to better understand the pathological processes of AD in obesity.

The unbiased skin lipidomics results showed that the most significant changes in the skin of the HF + AD mice were associated with AA metabolism. Consistently, transcriptomics analysis revealed a significant increase in *Pla2g4*, which is involved in breaking down phospholipids to generate AA, and up-regulated expression of genes related to the AA pathway in the HF + AD state. Additionally, other phospholipases, such as *Plcb1* and *Plcg1*, connecting atopic inflammatory responses with lipid metabolism, also exhibited significant alterations.

*Pla2g4a* encodes the only PLA2 subtype that exhibits marked substrate specificity for AA-containing PLs and produces two important inflammation mediators: AA and LPLs [24,25]. Nakano et al. [26] demonstrated that AA released from mast cells plays an important role in enhancing IgE receptor (FceRI)-mediated signal transduction, degranulation, and cytokine release. In addition, AA-derived metabolites are key players in allergic diseases [27]. Additionally, LPLs, produced by PLA2, play a crucial role in allergic diseases. LysoPC stimulates leukocyte activation, T-lymphocyte chemotraction, and histamine release from mast cells [28,29,30]. It also serves as a precursor to PAF, a potent inflammatory mediator [31]. Furthermore, lysophosphatidic acid (LPA) activates histamine release from mast cells and skin fragments [32]. Therefore, the inhibition of phospholipase A2 has been proposed as a potential therapy for treating inflammatory skin diseases [33].

In this study, we hypothesized that increased levels of AA in the skin might be related to changes in circulating lipids in the HF + AD group. Therefore, we analyzed the relationship between AA and its metabolites in the plasma and skin. The PLs containing FA 20:4, FA 24:4, and FA 24:5 in plasma exhibited positive associations with various lipids containing FA 20:4, FA 24:4, and FA 24:5 in the skin, whereas TGs containing FA 20:4, FA 24:4, and FA 24:5 exhibited negative associations with lipids containing FA 20:4, FA 24:4, and FA 24:5 in the skin. Furthermore, levels of the lipid transporter gene, *Scarb1*, responsible for transporting phospholipids [34], were found to be significantly increased in the HF + AD mice.

To further investigate the metabolism of transported fatty acids and phospholipids beyond their hydrolysis by phospholipase, we analyzed genes associated with phospholipid metabolism. Particularly, we examined the expression of the *AGPAT* family involved in acyltransferase activity in PL de novo synthesis and remodeling pathways. The results showed an increase in the levels of *Agpat6*, playing a role in *GPAT* activity rather than *AGPAT* activity [19], in the HF + AD group. Additionally, there was a significant increase in the expression of *Agpat3*. Notably, *Agpat3* exhibits acyltransferase activity in de novo synthesis and remodeling pathways, particularly when AA is present as an acyl donor for various LPLs, including LPA, LPC, lysophosphatidylserine (LPS), and lysophosphatidylinositol [20]. These findings suggest that in the HF + AD condition, AA-containing PLs may be elevated, resulting in the upregulation of *Pla2g4a* and a more stimulated AA pathway that triggers inflammatory responses (Figure 6G).

The translocated or remodeled PLs were also hydrolyzed by phospholipase C, whose levels increased in the HF + AD model, and generated diacylglycerol (DG) and inositol trisphosphate (IP3), which served as second messengers in cell signal transduction [35]. We also observed higher levels of DGs, including AA, in the HF + AD group than in the AD and sham control groups (Supplementary Figure S1) and found up-regulated DG kinase (*Dgk*) responsible for phosphorylating DG to regenerate PA [36]. However, the expression of *Lpin1*, *Cds1*, and *Cds2,* crucial for generating DG and CDP-DG from PA and playing essential roles in TG and PL de novo synthesis, was significantly decreased in the HF + AD group [37,38]. These results suggest a potential accumulation of PA in the HF + AD state, which has deleterious effects, including the stimulation of proinflammatory cytokine secretion, as well as the generation of nitric oxide and prostaglandin E2 in macrophages [39]. Moreover, it modulates the Akt-dependent activation of the mammalian target of rapamycin-p70 S6 kinase 1 pathway, contributing to the induction of inflammatory mediators [39,40]. In line with these previous results, we confirmed the alteration of PI3-Akt pathway and up-regulated expression of IL-6 in HF + AD compared with AD. In addition, we verified that these genes in phospholipid metabolism and AA levels were restored by AD treatment.

Phospholipids play a critical role in maintaining the integrity of the epidermal permeability barrier [41,42]. However, in the context of atopic stimulation, an increase in the levels of circulating arachidonic acid (AA) and AA-containing phospholipids may lead to excessive activation of phospholipase when they translocate to the skin. This excessive activation contributes to the immune response, potentially worsening atopic symptoms. Specifically, an imbalance in phospholipid metabolism, resulting in heightened AA and phosphatidic acid (PA)-mediated pathways in the skin of individuals with both high fat (HF)-diet-induced obesity and atopic dermatitis (AD), could exacerbate AD symptoms. Addressing this imbalanced phospholipid metabolism could offer a therapeutic approach to alleviating the exacerbated symptoms of AD induced by HF-diet-induced obesity. While our study sheds light on the interplay between lipid metabolism and inflammation in the HF + AD animal model, it may have limitations in terms of fully exploring the underlying molecular mechanisms. Further experiments and analyses are required to comprehensively elucidate these mechanisms in in vitro assays. Additionally, it is necessary to conduct further clinical studies to confirm our findings regarding dysregulated lipid metabolism as a potential therapeutic target, especially in regard to alleviating exacerbated symptoms of atopic dermatitis induced by HF-diet-induced obesity.

## 4. Materials and Methods

### 4.1. Animal Studies

Five-week-old male C57BL/6J mice were obtained from Saeron Bio Co. and housed in specific pathogen-free conditions. Mice were fed either a standard diet (sham) or a high-fat diet (HFD, D12492, Research Diets, New Brunswick, NJ, USA) for 10 weeks. AD-like lesions were induced using 0.5% DNCB solution applied thrice weekly for 6 weeks [43]. At the end of the experiment, mice underwent a 12 h fast before blood and tissue samples were collected. We performed euthanasia using anesthetic (240 mg/kg 2,2,2-tribromoethanol) for ethical considerations after concluding the experiment. Blood was collected in prechilled tubes containing 75 USP of heparin, and plasma was separated via centrifugation at 3000× *g* for 20 min at 4 °C. Additionally, skin tissues were collected, and some were fixed in 10% neutral-buffered formalin for histopathological examination, while others were rapidly frozen at −80 °C for RNAseq analysis. Prior to analysis, we utilized random sampling for each analysis due to a lack of samples. Further, the data points that fell outside the measurement confidence interval have been excluded.

### 4.2. Histological Analysis

Samples of equal size were taken from ears fixed in 10% neutral-buffered formalin for 24 h at 4 °C. Paraffin-embedded tissue sections (3–4 μm thick) were stained with hematoxylin and eosin (H&E). The sections were stained with toluidine blue to detect mast cells in the ears. The histological profiles of individual cross-sections of the samples were observed under a light microscope (Eclipse 80i; Nikon, Tokyo, Japan). To observe more detailed changes, the total thicknesses of the ear including cartilage, mean ear exterior epidermal thicknesses (μm), and number of inflammatory cells infiltrating the ear dermis (cells/mm2) were calculated under H&E staining using a computer-assisted image analysis program (iSolution FL ver 9.1, IMT i-solution Inc., Vancouver, British Columbia, Canada). In addition, mean mast cell numbers on the ear dermis (cells/mm2) under TB staining were also measured using a computer-assisted image analysis program according to previous methods with some modifications [44,45,46].

### 4.3. Measurement of Plasma Immunoglobulin E (IgE) and Interleukin-4 (IL-4)

The concentration of total IgE in plasma was measured using an enzyme-linked immunosorbent assay kit (Bethyl Laboratories Inc., Montgomery, TX, USA) according to the manufacturer’s instructions. For the analysis of IL-4 levels, plasma samples were assessed using the Bio-Plex Pro Mouse Cytokine Th1/Th2 Assay Kit (Bio-Rad Laboratories, Hercules, CA, USA). Additionally, the limits of detection (LLODs) and high limits of detection (HLODs) for various mediators, including IgE and Th1/Th2 mediators, are provided below (Table 2).

### 4.4. Lipidomics

For lipid analysis, skin (50 mg) and plasma (50 μL) samples were extracted from all mouse groups. Skin samples underwent homogenization (Precellys 24; Bertin Technologies, France) with 50% methanol and chloroform, while plasma samples were mixed with chloroform:methanol (2:1, *v*/*v*) and water. After centrifugation, the organic layer (lower phase) was dried and diluted for analysis. Lipid profiling was performed using ultra-high-performance liquid chromatography (UPLC; Acquity™ IClass; Waters, MA, USA) combined with quadrupole time-of-flight mass spectrometry (Q-TOF MS; Triple TOF™ 5600; Sciex, Toronto, ON, Canada) as previously described [47].

UPLC/Q-TOF MS data were processed using MS-Dial (RIKEN, Japan), as described by Lee et al. [48]. To ensure system stability, quality control (QC) samples were employed, which comprised a pool of all samples. These QC samples were analyzed every 10 runs. Features were identified as peaks with intensities greater than 5 times that of blank samples. Additionally, features with coefficients of variation below 20 in the QC samples were selected to assess detection precision. Lipid metabolites were identified based on an m/z and ms/ms spectral match using in silico-based metabolite databases, including Metlin (https://metlin.scripps.edu (accessed on 23 February 2024)), LIPID MAPS (www.lipidmaps.org (accessed on 23 February 2024)), MassBank of North America (http://mona.fiehnlab.ucdavis.edu (accessed on 23 February 2024)), and the Human Metabolome Database (HMDB, www.hmdb.ca (accessed on 23 February 2024)).

Partial least squares-discriminant analysis (PLS-DA) was performed using SIMCA-P+ software (version 14.0; Umetrics, Umea, Sweden), validated using permutation and CV-ANOVA tests. Lipid pathway enrichment analysis was performed using LIPEA (https://lipea.biotec.tu-dresden.de/home (accessed on 23 February 2024)).

### 4.5. RNA-Sequencing (RNA-Seq)

Total RNA was extracted from mouse skin tissue using TRIzol reagent (Invitrogen, Carlsbad, CA, USA) and assessed for quality using an Agilent 2100 Bioanalyzer (Agilent Technologies, Amstelveen, The Netherlands). RNA Integrity Number (RIN) values, as required by MIAME (Minimum Information About a Microarray Experiment) guidelines, are provided in Supplementary Table S1. RNA libraries were prepared with the QuantSeq 3′ mRNA-Seq Library Prep Kit (Lexogen, Inc., Vienna, Austria) and sequenced using a NextSeq 500 system (Illumina, Inc., San Diego, CA, USA). Read count data were normalized using edgeR [49]. Enrichment analysis of gene sets was performed using MetaboAnalyst 5.0 (https://www.metaboanalyst.ca (accessed on 23 February 2024)). Functional enrichment of DEGs (*p* < 0.05, >2.0-fold changes) was analyzed using ClueGO/CluePedia (2.5.9) plugin in Cytoscape (3.9.1), with Cerebral plugin used to arrange biological networks based on localization information [50].

### 4.6. Statistical Analysis

Statistical analyses were conducted using GraphPad Prism version 9 (GraphPad Software, La Jolla, CA, USA), with significance set at *p* < 0.05. Data are presented as mean ± standard error of the mean. One-way or two-way ANOVA followed by Tukey’s test were used for three-group comparisons, while the Kruskal–Wallis test with Dunn’s multiple comparison test was employed for non-Gaussian distribution data. Two-group comparisons were performed using a two-sided t-test or the Mann–Whitney U test for non-Gaussian distribution data.

## 5. Conclusions

In summary, our study has demonstrated that a high-fat (HF) diet can exacerbate symptoms of atopic dermatitis (AD) by disrupting lipid metabolism in the skin. Specifically, we found that an imbalance in phospholipid metabolism in the skin of HF-fed mice with AD may contribute to the increased inflammatory response observed in this group. This suggests a potential mechanism by which obesity could worsen AD symptoms. Addressing this imbalanced phospholipid metabolism could potentially provide a therapeutic target to alleviate the exacerbated AD symptoms induced by HF diet-induced obesity.

## Figures and Tables

**Table 1:** The result of lipid pathway enrichment analysis.

Pathway Name	Converted Lipids	*p*-Value	Benjamin Correction	Bonferroni Correction
Arachidonic acid metabolism	100	8.52 × 10^−7^	5.11 × 10^−6^	5.11 × 10^−6^
Inflammatory mediator regulation of TRP channels	28.57	0.02	0.05	0.11
PPAR signaling pathway	14.29	0.06	0.12	0.39
Vascular smooth muscle contraction	14.29	0.06	0.12	0.61
Aldosterone synthesis and secretion	14.29	0.1	0.12	0.61
Serotonergic synapse	14.29	0.36	0.36	1

**Table 2:** Limits of Detection (LLODs) and High Limits of Detection (HLODs) for basic mediators (IgE) and Th1/Th2 mediators in IL–4 measured using the Bio–Plex assay.

	Mediators	LLODs	HLODs
Basic	IgE	1.37 ng/mL	1000 ng/mL
Th1/Th2	IL–4	0.36 pg/mL	5947 pg/mL

## Data Availability

Raw sequences and processed data were deposited in the NCBI Gene Expression Omnibus (GEO, https://www.ncbi.nlm.nih.gov/geo/ (accessed on 10 March 2023)) with accession number GSE227042. Data presented in this study are available upon request from the corresponding author.

## Supplementary Materials

The following supporting information can be downloaded at https://www.mdpi.com/article/10.3390/ijms25084143/s1.
