# Supplementary material for: Integrated Omics Analysis Uncovers the Culprit behind Exacerbated Atopic Dermatitis in a Diet-Induced Obesity Model
Source: Int J Mol Sci. 2024 Apr 9;25(8):4143. doi: 10.3390/ijms25084143 (PMC11050523; doi:10.3390/ijms25084143)
Supplement: Supplementary file 1 [file ijms-25-04143-s001.zip › ijms-2913025-supplementary.pdf]

Integrated omics uncover the culprit of exacerbated atopic dermatitis in a diet-induced obesity model

You Mee Ahn[1,†], Jeeyoun Jung[1,†,*], So Min Lee[1]

[1] KM Science Research Division, Korea Institute of Oriental Medicine, Daejeon 34054, Republic of Korea
* Correspondence: jjy0918@kiom.re.kr; Tel.: +82-42-868-9272
† These authors contributed equally to this work

Supplementary Table S1. Description of RNA quality assessment.

No.	Sample	Group	Nano-drop (ND 2000)				Bioanalyzer (Nano)	
			ng/µl	Total (µg)	OD 260/280	OD 260/230	Ratio (28s/18s)	RIN
1	1_1	Sham	133.4	1.601	2.00	1.94	0.4	2.4
2	1_2		184.4	2.212	1.95	1.82	0.6	2.4
3	1_3		425.0	5.100	2.03	2.09	0.6	2.3
4	1_4		198.1	2.377	2.05	2.02	0.8	2.5
5	2_1	AD	1701.9	22.125	2.11	2.19	0.1	2.5
6	2_2		3316.6	43.116	2.01	2.07	0.6	2.5
7	2_3		4220.4	54.865	1.62	1.67	0.1	2.5
8	2_4		4192.6	54.504	1.62	1.69	0.1	2.4
9	2_5		4258.3	55.358	1.50	1.58	0.1	2.4
10	3_1	HF+AD	4285.8	55.715	1.52	1.60	0.2	3.0
11	3_2		4113.7	53.478	1.72	1.80	0.3	2.8
12	3_3		4343.1	56.460	1.29	1.39	0.2	3.1
13	3_4		3871.3	50.327	1.86	1.93	0.1	2.3
14	3_5		4302.3	55.930	1.32	1.39	0.2	2.7
15	3_6		4130.5	53.697	1.74	1.80	0.1	2.5
16	3_7		4242.8	55.157	1.58	1.62	0.2	3.2

OD, optical density; RIN, RNA integrity number.

Supplementary Figure S1

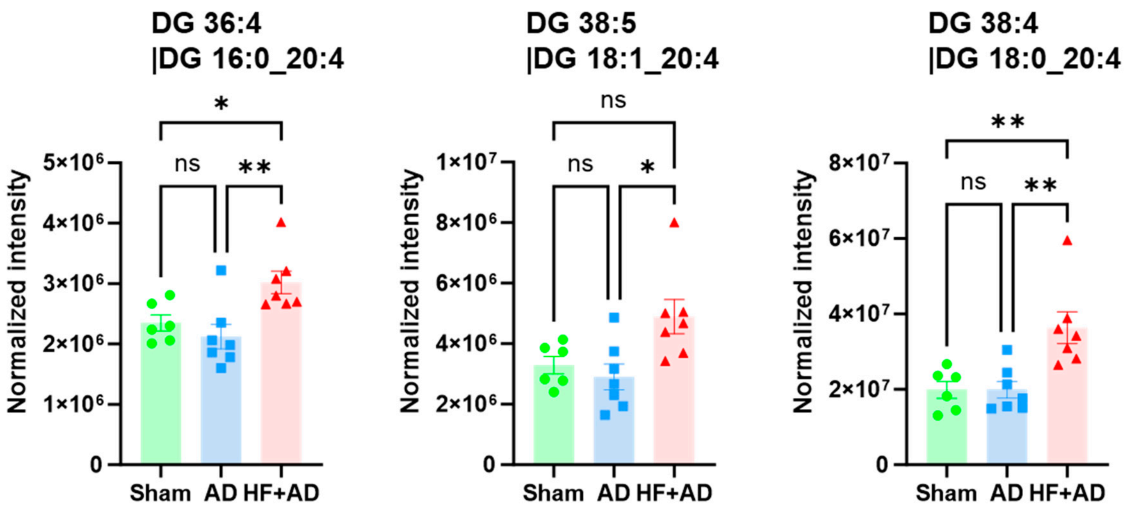

Figure S1. AD influences skin lipid metabolism in HF+AD mice. The alteration of DGs in 36:4, 38:5, and 38:4 including arachidonic acid (FA 20:4). * $p < 0.05$, ** $p < 0.01$ estimated using one-way ANOVA with Tukey's multiple comparisons tests. HF, high-fat; AD, atopic dermatitis, DG, diglyceride; ANOVA, analysis of variance.
